# Supplementary material for: Mixed Depression: A Mini-Review to Guide Clinical Practice and Future Research Developments
Source: Brain Sci. 2022 Jan 11;12(1):92. doi: 10.3390/brainsci12010092 (PMC8773514; doi:10.3390/brainsci12010092)
Supplement: Supplementary file 1 [file brainsci-12-00092-s001.zip › brainsci-1533979-supplementary.pdf]

Supplementary material

Table S1. Definition of mixed depression and inclusion criteria used in the main guidelines.

**Canadian Network for Mood and Anxiety Treatments (CANMAT) and International Society for Bipolar Disorders (ISBD)**	Mixed depression in bipolar patients type I and II, according to:<br><br>- DSM-5: Ratings and recommendations are based predominantly on studies of participants who met DSM-5 proxy criteria but also include studies of those with manic episodes with fewer than three concurrent depressive symptoms, or symptom rating scale cut-offs consistent with syndromal manic episode and subsyndromal depressive symptoms.<br>- DSM-IV: Ratings described are based on studies in participants with DSM-IV mixed episodes, or similar definitions.
**Word Federation of Societies of Biological Psychiatry (WFSBP)**	Acute treatment of a bipolar depressive episode with ≥3 hypomanic or manic symptoms as defined by DSM-5.
**International College of Neuropsychopharmacology (CINP)**	Mixed episode in bipolar disorder.
**Royal Australian and New Zealand College of Psychiatrists (RANZCP)**	Mixed depression in mood disorders. A mixed state is defined as any mood state in which symptoms traditionally described as belonging to either mania or depression are present alongside symptoms conventionally thought of as belonging to the other pole.
**Stahl's Guidelines**	The guideline recommendations throughout this article are made in reference to DSM–5 rather than DSM–IV descriptions and criteria.

Legend: DSM=Diagnostic and statistical manual of mental disorders
